# Supporting Malaria Diagnosis Using Deep Learning and Data Augmentation

**DOI:** 10.3390/diagnostics14070690

**Published:** 2024-03-25

**Authors:** Kenia Hoyos, William Hoyos

**Affiliations:** 1 Human Clinical Laboratory, Social Health Clinic, Sincelejo 700001, Colombia; kmhoyosgonzalez@gmail.com; 2 Sustainable and Intelligent Engineering Research Group, Cooperative University of Colombia, Montería 230002, Colombia; 3 R&D&I in ICT, EAFIT University, Medellín 050022, Colombia; 4 Microbiological and Biomedical Research Group of Cordoba, University of Córdoba, Montería 230002, Colombia

**Keywords:** malaria, deep learning, artificial intelligence, *Plasmodium*, diagnosis

## Abstract

Malaria is an infection caused by the *Plasmodium* parasite that has a major epidemiological, social, and economic impact worldwide. Conventional diagnosis of the disease is based on microscopic examination of thick blood smears. This analysis can be time-consuming, which is key to generate prevention strategies and adequate treatment to avoid the complications associated with the disease. To address this problem, we propose a deep learning-based approach to detect not only malaria parasites but also leukocytes to perform parasite/μL blood count. We used positive and negative images with parasites and leukocytes. We performed data augmentation to increase the size of the dataset. The YOLOv8 algorithm was used for model training and using the counting formula the parasites were counted. The results showed the ability of the model to detect parasites and leukocytes with 95% and 98% accuracy, respectively. The time spent by the model to report parasitemia is significantly less than the time spent by malaria experts. This type of system would be supportive for areas with poor access to health care. We recommend validation of such approaches on a large scale in health institutions.

## 1. Introduction

Malaria is a potentially fatal disease caused by parasites of the genus *Plasmodium*. The disease is transmitted to humans by the bite of infected female mosquitoes of the genus *Anopheles* [1]. According to the World Health Organization (WHO), by 2021, 247 million cases were estimated worldwide, while 619,000 deaths were reported for the same year [2]. Diagnosis and treatment are key factors in reducing morbidity and mortality rates. Early diagnosis and treatment reduce the risk of death from malaria [3]. There are different methods for the diagnosis of malaria, among which are the following: (1) the gold-standard method consisting of microscopic examination of stained blood smears (thick drop), which requires a lot of time and expertise [4]; (2) rapid diagnostic tests, which are inexpensive and useful in resource-poor settings [5]; (3) polymerase chain reaction, which shows high sensitivity and specificity, with its time consumption being its main disadvantage [6]; and (4) flow cytometry, which is a method based on detecting and counting cells using a special laser [7]. The time it takes is a limitation for the rapid diagnosis of the disease; therefore, it is necessary to develop tools or strategies to improve the speed and accuracy of the results.

In recent years, computational strategies have been developed to help improve malaria diagnosis mainly by supporting decision making with respect to microscopic examination. The literature reports several papers using fine blood smear images [8,9,10,11]. However, few studies have been developed using thick blood smears [12,13,14]. One of the drawbacks of thick blood smears is the concentration of the samples, which hinders the process of object recognition in the images. In addition, despite great efforts, no strategies have been developed that allow the detection of both parasites and leukocytes to perform the estimation of parasite density necessary to report the results. It is important to remember that malaria diagnosis consists not only of parasite detection but also of parasite density expressed in parasites per ul of blood, which, according to diagnostic guidelines, uses the leukocytes present [15]. Thus, it is crucial to develop approaches that allow not only the detection of parasites but also the detection of leukocytes, and with the results of the detection, generate a report of the parasite density.

Based on the needs found, we propose an automated approach for the detection of parasites and leukocytes in thick-drop images to aid in the diagnosis of malaria. The main contributions of this work are as follows: (1) a deep learning (DL)-based model to detect malaria parasites and leukocytes in thick-drop smear images; (2) a model that performs estimation of parasite density based on malaria diagnostic guidelines; and (3) a model that performs malaria detection faster than medical experts. We compared the performance of our model with the performance of malaria experts, demonstrating high agreement between the results. Finally, we measured the time to read and count parasites in image packets with favorable results for the model versus malaria experts.

The remainder of this paper is organized as follows: Section 2 presents the related work regarding studies using artificial intelligence (AI) for malaria detection. Section 3 shows a theoretical background related to malaria and DL. Section 4 describes the methodology used for the fulfillment of the objective. Section 5 presents the results of the proposed approaches. Section 6 discusses the results and compares them with previous work. Finally, Section 7 concludes, describes the limitations and recommends future work.

## 2. Related Work

The development of models using DL techniques applied for malaria parasite detection from blood smear images has increased in recent years [16]. Conventionally, malaria parasite detection and parasite density counting is mainly performed on two types of preparation: thin blood smear and thick drop smear [17]. In the following, we present some studies that used images of each type of preparation.

### 2.1. Detection of Malaria Parasites in Thin Blood Smear Images

There is a wide trend of publications in the literature that have focused on red blood cell parasite detection in thin blood smears [18,19,20,21,22]. For example, Silka et al. [18] developed a convolutional neural network architecture to detect malaria from images of thin peripheral blood smears; the model obtained an accuracy of 99.7%. However, the model did not count leukocytes and does not calculate parasite density. These two aspects are important for establishing the best therapeutic strategy for malaria patients. Marques et al. [19], employed a decision support system for malaria detection based on the EfficientNet architecture for the classification of red blood cells into parasitized and non-parasitized groups with an accuracy of 97.74%. Like the work of Silka et al., the authors did not take into account leukocytes, which are necessary for the calculation of parasite density. The report of the number of parasites per μL/blood is necessary because it allows to know the severity of the disease and the response to treatment. Loh et al. [20] developed a DL model based on a convolutional neural network called Mask R-CNN, in order to detect and quantify red blood cells and reticulocytes parasitized by *P. falciparum* at different stages. The model achieved an accuracy of 94.57% in the detection of infected cells with an error of 0.55%, yielding a quantitative value of cells, parasite stages, and a percentage of parasitemia; the study obtained good results of readings compared to reading by experts, with an average analysis time of 4 s per image. Vijayalakshmi et al. [21] developed a deep neural network model to identify the malaria parasite *P. falciparum* using a transfer learning approach by unifying the Visual Geometry Group (VGG) network and support vector machine (SVM). The pre-trained VGG facilitates the role of the expert learning model and SVM as a domain-specific learning model. The VGG-SVM model was compared with CNN, resulting in 93.1% classification accuracy in malaria identification, with superior results to CNN. This unification facilitates the ability to use prior knowledge of VGG as model parameters to learn and SVM to classify malaria images with infected and uninfected cells. Alkhaldi et als. [22] developed a convolutional neural network-based model with thin blood smear images to diagnose malaria disease. An overall accuracy of 97% was achieved. The training dataset contained 43,827 photographs of healthy and malaria parasite-infected red blood cells. The results are good; however, they do not perform detection and counting of parasites along with leukocytes to calculate parasite density, which is important for diagnosis and treatment guidance of the disease.

### 2.2. Detection of Malaria Parasites in Thick Blood Smear Images

Despite the high number of studies using images of thin blood smears, the WHO recommends that microscopic diagnosis be performed on thick smears because it increases the probability of an accurate diagnosis due to a higher concentration of the sample [5]. Several decision support systems using models trained and evaluated with thick-drop imaging and DL models have shown good results [23,24,25,26]. Yang et al. [23] developed an automated system for cell phones to detect and classify malaria parasites using the intensity-based Iterative Global Minimum Screening method and an RNC. The system was trained with full images with resolutions of 3024 × 4032. The evaluation results yielded an accuracy of 93.46%, accuracy of 94.25%, and specificity of 94.33%. The system had the ability to measure detection and classification time with an average of 10 s per image. De Souza et al. [24] developed a two-stage system, where the first stage classifies pixels with random forest and multilayer perceptron, and then the obtained patches are classified with a CNR. The performance on four sets of images with patches of different sizes was evaluated, and the best accuracy was obtained in the recognition of large patches with a range between 91.71% and 93.14%. Regarding the time needed to evaluate a 1044 × 1388 pixel image, it was approximately 6 s. Chibuta et al. [25] used a modification of YOLOv3 on two datasets with different resolutions, achieving accuracies of 99.07% and 97.46%. The stopping time depends on the pixels of the input image, for example, in images with 800 × 800 resolution, it was 0.42 s. The authors showed that reducing the input image size at test time affects the accuracy and precision, while increasing the input size increases the computational cost. Manescu et al. [26] developed a detection system called DeepMCNN in order to quantify *P. falciparum* parasites, white blood cells, and parasitemia determination according to WHO recommendations. The model obtained a sensitivity of 92%, a specificity of 90%, and an accuracy of 91%, finding that in patients with high parasite loads, there was concordance with the experts; however, in low and medium parasitemia, the result of parasite densities was overestimated.

## 3. Theoretical Framework

In this section, we show the theoretical framework on the main aspects related to malaria, such as its etiological agent, clinical manifestations, and conventional diagnosis. In addition, we present related concepts about DL and the YOLO algorithm.

### 3.1. Malaria

Malaria is a parasitic disease caused by protozoa of the genus *Plasmodium*, which are inoculated into the host by the female *Anopheles* mosquito during the feeding process [1]. Five species cause infections in humans: *P. falciparum*, *P. vivax*, *P. malariae*, *P. ovale*, and *P. knowlesi*, where the highest infection rates have been associated with *P. falciparum* and *P. vivax* [6,27]. This disease is characterized by a clinical picture with high fevers, sweating, and chills. Malaria may be suspected clinically by signs and symptoms, epidemiologically by the patient’s travel history or area of residence, but the definitive diagnosis is made on the basis of laboratory tests that must demonstrate the presence of malaria parasites or their components [28]. Rapid diagnosis and timely treatment of the disease is crucial to reduce complications and death of infected patients, being two important components for worldwide strategies for malaria control and elimination [29].

#### 3.1.1. Clinical Manifestations of Malaria

The clinical diagnosis of malaria is difficult because the signs and symptoms of the disease are nonspecific and similar to other febrile infections [28]. Clinical manifestations can range from asymptomatic, mildly symptomatic, to severe, life-threatening illnesses, and are classified into two clinical forms: uncomplicated malaria and complicated malaria [30]. Uncomplicated malaria is characterized by the sudden onset of fever, general malaise, chills, headache, muscle and joint pain, and sweating; febrile paroxysms may occur according to the infecting parasite species. Complicated malaria presents with vital organ dysfunction, plasma extravasation leading to shock, cellular hypoxia, metabolic acidosis, severe anemia, acute renal failure, pulmonary edema, and severe organ and system involvement [28,30]. In endemic areas, malaria should be suspected in patients with fever ≥ 37.5 °C, children presenting with palmar pallor or hemoglobin concentration better than 8 g/dL [31] because severe anemia leads to increased infant mortality and is a leading cause of hospital admissions in Africa [32].

#### 3.1.2. Malaria Diagnosis

Microscopic examination, rapid diagnostic tests and molecular tests are the three most commonly used methods for malaria diagnosis. Microscopic examination remains the standard test for the diagnosis of malaria [33] because it allows the quantification and identification of the infecting species [31]. Two methodologies can be used to perform the examination: (i) thick drop smear: two drops of blood are used on a slide where the objective is to concentrate the sample, which is going to allow the detection and quantification of the parasite; and (ii) peripheral blood smear: a drop of blood is spread on the slide to obtain a thin layer and allow the identification of malaria species [17]. The slides are stained with stains derived from Romanovsky such as Giemsa, Wright or Field, and are observed with a 100× objective [6,34]. A minimum of 200 microscopic fields must be reviewed, without finding parasitic forms to consider the sample negative. In the case of positive samples, a count of parasites present in 200 leukocytes is performed to subsequently calculate the total count with the number of leukocytes of the patient; if the result is not available, the average leukocyte value of 8000 leukocytes/µL is taken [5]. Microscopy has several advantages, among which are low costs, good sensitivity, the detection of parasitemia, and the differentiation of parasite species and stages; it allows patient control by monitoring the response to treatment and can also help diagnose other diseases [5]. However, microscopy has disadvantages such as the lack of experience of microscopists in the diagnosis of malaria by not correctly detecting and identifying the species of *Plasmodium* spp., mainly in non-endemic areas [27,35], heavy workloads that reduce the timeliness of reports, difficulty with the costs of maintaining the facilities of health services, and the lack of quality assurance programs that ensure the correct use of reagents, assembly of tests and good condition of the equipment [5].

Rapid diagnostic tests (RDTs) are immunochromatographic tests, which allow the detection of parasite antigens such as histidine-rich protein II (HRP-II), lactate dehydrogenase (pLDH) or aldolase [36,37]. The blood sample flows through a membrane containing specific antimalarial antibodies that bind to parasite antigens present on the patient’s red blood cells [38]. RDTs contribute to malaria diagnosis by offering an alternative in areas where reliable microscopic diagnosis is not available, decrease reporting time since they generally take 20 min, do not require highly trained personnel to perform and interpret the tests, are relatively inexpensive, do not require electricity or laboratory facilities, and are easy to perform in remote rural settings [39].

With the advance of molecular biology, different technologies have been created that allow the development of new strategies for the diagnosis and characterization of the malaria parasite, such as polymerase chain reaction (PCR), loop-mediated isothermal amplification (LAMP), microarrays, mass spectrometry (MS), and flow cytometry assay (FCM) techniques [6]. The most widely used technique is PCR, which allows the detection of parasite nucleic acids and has high specificity and sensitivity, mainly in cases of low parasitemia or mixed infections [40]. Despite the benefits of PCR, it has some disadvantages; for example, it requires trained personnel for the procedure, it is not available in settings with first-level health institutions or rural areas, and it presents delays in the results needed to establish the timely diagnosis of the infection [28].

### 3.2. Introduction to DL

DL is a machine learning approach that uses algorithms based on a deep neural network model. These algorithms attempt to simulate human learning behavior using as an example brain synapses, in which neurons transmit information to each other [41]. This type of algorithm performs on large datasets, mainly unstructured data such as images, videos and audios. The basic architecture used in DL is based on convolutional neural networks, which are inspired by the architecture of the primary visual cortex of a biological brain [42].

#### Convolutional Neural Networks

A convolutional neural network is a type of artificial neural network which uses deep layers to find functional dependencies between input variables and labels. A CNN consists of three types of layers: convolutional, clustering, and fully connected. Convolutional layers are filters used to extract and map features [43]. Pooling layers are used to reduce the dimensionality and thus the parameters of the input features. Two of the most commonly used pooling processes are max and average. In this way, pooling extracts representative features from the input variables while keeping the most important information, reduces the computational overhead, and aids in the reduction in overfitting [44]. Finally, the fully connected layers are those that use the information from the previous feature analysis layers and optimize weights to generate the correct prediction label. Within these layers, we find the output layer, which provides the probabilities of each label in classification problems [45]. This type of network can be used for prediction, classification, object detection in images, videos, and audio analysis, among other applications [46].

### 3.3. YOLO Algorithm

YOLO is a DL-based object detection algorithm that has gained popularity in the field of computer vision. Its innovative approach focuses on accurate and fast object detection in real-time images and videos [47]. Unlike previous approaches that split detection into multiple stages, YOLO tackles the problem as a single step, making it extremely efficient in terms of execution time. This makes it a powerful choice for a variety of applications, such as object detection in autonomous vehicles [48], surveillance [49], and video analysis [50].

Different versions of YOLO have been developed, from version 1 to version 9. However, version 8 is the most robust and stable version that has been developed to date. The architecture of YOLOv8 is similar to YOLOv5 with some slight modifications: (1) change of module C3 to module C2f; (2) changes in the initial convolution of the backbone (6 × 6 to 3 × 3); (3) the removal of convolutions 10 and 14 from the YOLOv5 architecture; (4) the replacement of the initial convolution in the bottleneck from 1 × 1 to 3 × 3; and (5) the removal of the object branch using the decoupled head [51]. Considering its ability to detect objects and that it is the latest version of the approach, we used YOLOv8 to detect malaria parasites and leukocytes in thick-drop images.

## 4. Materials and Methods

**Figure 1:**
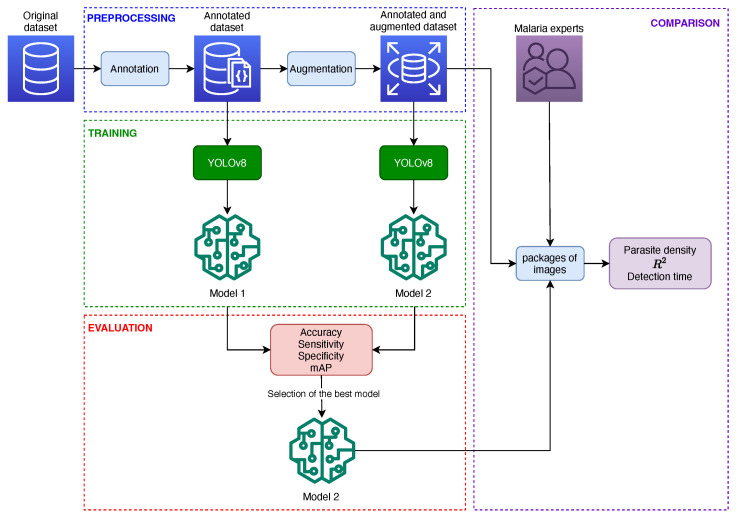
Methodological framework of this research.

This section describes the methodology used for the development of the study. Figure 1 represents the methodological framework and summarizes the methodological process. Initially, we described the dataset and then performed the annotation and data augmentation process to increase the size of the dataset. Subsequently, we built the detection models and compared them with respect to the dataset used. Then, we selected the best model for the estimation of parasite density and detection time evaluation. Finally, we compared the model results with the results generated by the malaria experts.

### 4.1. Dataset

**Figure 2:**
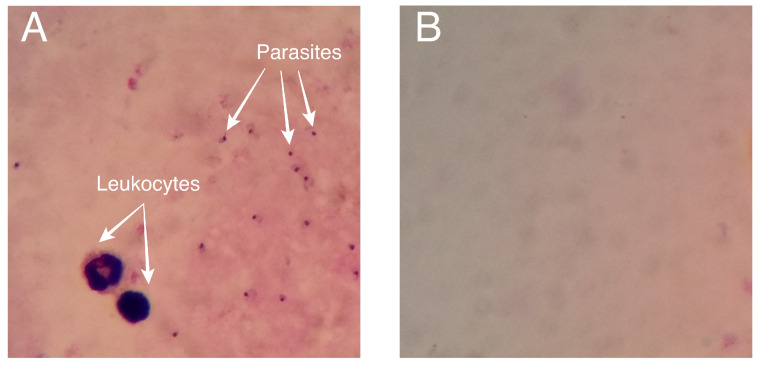
Thick blood smear images from the dataset: (**A**) positive for malaria with some parasites and leukocytes, (**B**) negative image for malaria.

The dataset used for this research was obtained from the Makerere AI Lab in Uganda [52]. The images contained in the dataset are hosted in an open access public repository and licensed under a Creative Commons Attribution 4.0 International License. From the dataset, we selected 333 thick blood smear images corresponding to malaria positive and negative samples. The images have a resolution of 750 × 750 pixels. In Figure 2, we can see some positive and negative images with parasites and leukocytes extracted from the dataset.

### 4.2. Preprocessing

In this section, the process of dataset annotation and augmentation is presented. First, a process of annotation was carried out to identify objects in images using bounding boxes. Next, we used augmentation techniques to increase the dataset size to prevent overfitting and achieve better results.

#### 4.2.1. Annotation

**Figure 3:**
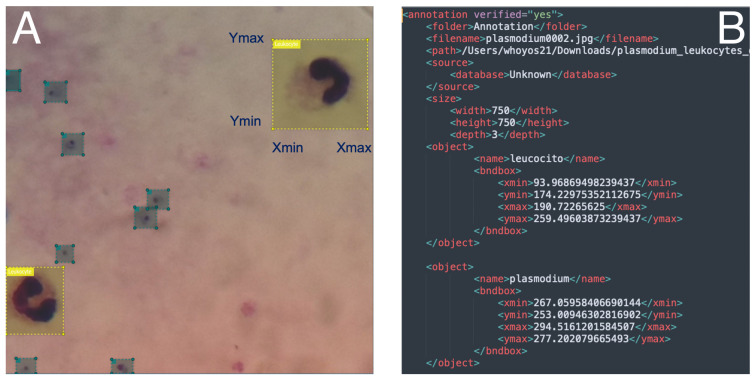
Example of labeling process of images. (**A**) indicates an image with green bounding boxes = parasites, yellow bounding boxes = leukocytes. (**B**) represents a file with locations of bounding boxes.

The dataset was labeled by one expert in malaria diagnosis. For this purpose, the Visual Object Tagging Tool (VoTT version 2.2.0) developed and maintained by Microsoft^®^ was used [53]. VoTT is a tool that allows the generation of datasets and validation of object detection models either in images or videos. This application was chosen because of its main features: (1) the ability to tag and annotate independent directories of images; and (2) the export of labels to Custom Vision Service (CNTK), Tensorflow (PascalVOC) or YOLO format for training an object detection model. The process consisted of two steps: (1) The expert labeled in a bounding box each of the elements in the image. Figure 3 shows an example of labeling an image with leukocytes and parasites. (2) At the end of labeling of all the images, a json file was exported with all the labels for the images. This file was used to feed the DL model.

#### 4.2.2. Data Augmentation

Data augmentation is a strategy to artificially generate data from original data. The objective is to increase the size of the dataset to avoid problems such as overfitting [54]. In this case, different perturbations are performed in the original images to obtain new images. In our study, we used the services of RoboFlow [55], which allowed to automatically generate images with different modifications. The following augmentation was applied to create 3 versions of each source image: (i) 50% probability of horizontal flip, (ii) 50% probability of vertical flip, (iii) equal probability of 90-degree rotations (none, clockwise, counter-clockwise), (iv) HSV color transformation, and (v) grayscale.

### 4.3. Model Training

We used 70% of the data for training and validation, which was carried out in CreateML, a machine learning platform developed by Apple^®^. The training was performed on a Macbook Air with M1 processor. This processor contains an 8-core GPU and a 16-core Neural Engine. The latter was optimized for training machine learning models and can perform 11 billion operations per second. The training was performed for 300 epochs.

### 4.4. Model Evaluation

In this section, we present the strategies used for the evaluation of the model both to assess its predictive capability and computational cost. The model evaluation was performed on 30% of the data.

#### 4.4.1. Evaluation of Predictive Capacity

We used accuracy, sensitivity, specificity, mAP and $R^{2}$ to evaluate the predictive performance of the model. Accuracy is calculated as the number of correctly detected instances over the total number of instances present. An instance is considered correctly classified if the Intersection over Union (IoU) > 0.5. Sensitivity is the proportion of positive instances that are correctly identified by the model, while sensitivity is the proportion of negative instances correctly predicted by the model. Finally, we used $R^{2}$ to measure the degree of agreement between the parasite report made by the model and the count made by the experts. The following equations define the metric used:

(1) $$Accuracy=\frac{TP+TN}{TP+FN+FP+TN}$$

where TP are the true positives, TN are true negatives, FN are false negatives, and TN are true negatives:

(2) $$Sensitivity:\frac{TP}{TP+FN}$$

(3) $$Specificity:\frac{TN}{TN+FP}$$

(4) $$mAP=\frac{1}{n}\sum_{i=1}^{n}AP\left(i\right)$$

where *n* represents the number of categories, and *AP* is the average precision of a certain class.

(5) $$R^{2}=\frac{\sum_{i=1}^{m}{\left({\hat{y}}_{i}-{\bar{y}}_{i}\right)}^{2}}{\sum_{i=1}^{m}{\left(y_{i}-{\bar{y}}_{i}\right)}^{2}}$$

where $y_{i}$ is the value reported by each expert, ${\hat{y}}_{i}$ is the value reported by the Model 2, and ${\bar{y}}_{i}$ is the mean of values reported by the experts.

#### 4.4.2. Evaluation of Reading and Reporting Time

Malaria diagnosis must be made quickly to define prevention and treatment strategies. In this study, we compared the time taken by the best model to make the diagnostic report with the time taken by three expert laboratory technicians. The objective was to determine if there were significant differences between the time spent by the model when is compared with experts. We used the Kolmogorov–Smirnov test with the Lilliefors correction to test the normality of the data, and the Kruskal–Wallis test to compare the mean time of the model with the mean of the experts. We performed Dunn’s test to evaluate pairwise comparisons between the model and each of the experts. The following is the hypothesis for the Kruskal–Wallis test:

- $H_{0}:{\tilde{y}}_{1}={\tilde{y}}_{2}=\cdots ={\tilde{y}}_{r}$
- $H_{1}:{\tilde{y}}_{i}\neq {\tilde{y}}_{i^{\prime }}$ for $i\neq i^{\prime }$

where $\tilde{y}$ is the median of the performance of each expert or model. After the comparison between the experts and model, the null hypothesis is rejected, and it is concluded that there are significant differences between the medians of the groups. However, it is not specified exactly which of them are different from the others. To know between which groups there are significant differences, Dunn’s test was used. The following hypothesis will be verified:

- $H_{0}:{\tilde{y}}_{i}={\tilde{y}}_{i^{\prime }}$ for $i\neq i^{\prime }$
- $H_{1}:{\tilde{y}}_{i}\neq {\tilde{y}}_{i^{\prime }}$ for $i\neq i^{\prime }$

## 5. Results

In this section, we present the results obtained from data augmentation, and the detection of malaria parasites and leukocytes. Additionally, we show the results of the estimation of parasite density using these two types of elements. Finally, we present the results related to the computational time of the model to detect the objects in the images.

### 5.1. Data Augmentation

**Figure 4:**
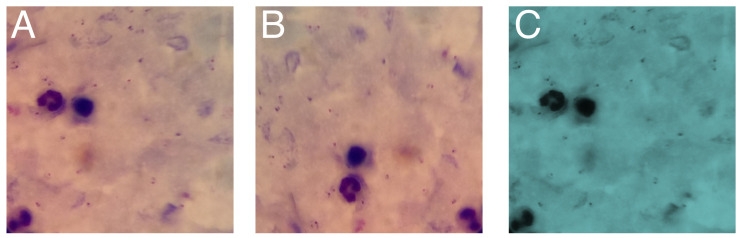
Examples of data augmentation from an original image. (**A**) represents an image of the original dataset. (**B**) represents an image with a 90° rotation. (**C**) represents an image with random HSV transformation.

It has been shown that increasing the size of the dataset for training DL models guarantees better performance [56]. For this reason, we used the data augmentation process. We used 222 images to generate 3 new images for each original image with the variations described in Section 4. At the end of the process, we obtained 666 images, which were used for training. Figure 4 shows an example of the variations generated with the data augmentation process.

### 5.2. Performance of the Models in Detecting Parasites and Leukocytes

Table 1 shows the performance of the models developed to detect parasites and leukocytes. In this case, we trained two models: (1) model trained with original data, and (2) model trained with augmented data.

Using the original dataset, the YOLOv8 algorithm was trained on 222 images for the detection of malaria parasites and leukocytes. In this case, the dataset had two classes: parasites and leukocytes. The model was tested on 30% of the images corresponding to 111 images. The results yielded 91% and 90% accuracy for parasites and leukocytes, respectively. Subsequently, using the augmented dataset, the YOLOv8 algorithm was trained on 666 images for the detection of malaria parasites and leukocytes and tested on the same test set. The results yielded an accuracy of 95% and 98% accuracy for parasites and leukocytes, respectively. In terms of mAP, higher values were obtained when using the augmented dataset for training. These results remained consistent for both parasite and leukocyte detection tasks. Similarly, the sensitivity and specificity results were higher when the dataset was increased (see Table 1).

### 5.3. Parasite Density Calculation

To determine the parasite density (parasites/μL of blood), we used the number of parasites and the number of leukocytes at the same time. The following WHO formula was used to calculate the parasite density [15]:

(6) $$PD=\frac{NPC\times 8000}{NLC}$$

where PD is the parasite density, NPC is the number of parasites counted, and NLC is the number of leukocytes counted. WHO recommends the value of 8000 as an estimated average of leukocytes when patient information related to the blood count is not available [15]. In addition to detection, we set out to evaluate the agreement between the parasite density reported by the experts and the parasite density generated by the Model 2, which performed the best. To achieve this, 50 images with replacement were randomly taken from the test dataset to obtain 30 packets of 50 images. The packets were provided to three malaria clinical experts assuming that each packet was a patient. At the same time, the image packets served as input to the Model 2 for the detection and quantification of parasite density. The following formula was used to establish a mean value representing the experts’ report:

(7) $$\bar{d}=\frac{1}{N}\sum_{i=1}^{N}E_{i}$$

where $\bar{d}$ is the average parasite density, *N* is the number of experts, and $E_{i}$ is the parasite density reported by expert *i*.

**Figure 5:**
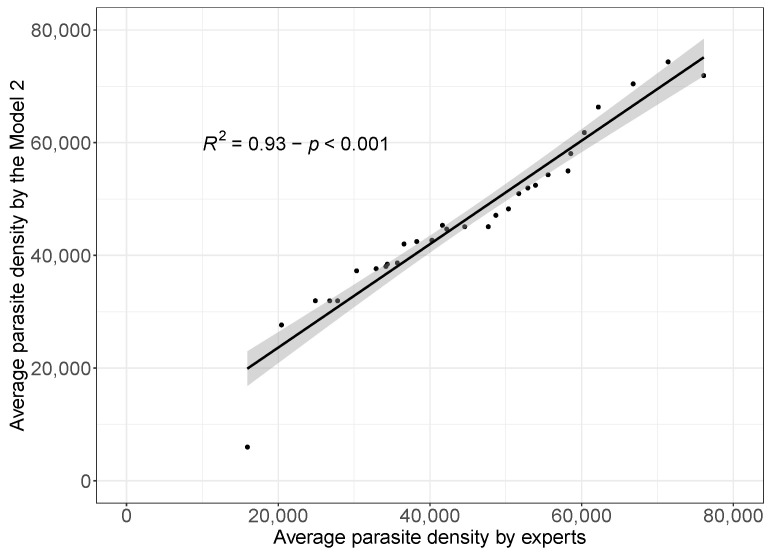
Scatterplot to visualize the agreement between the experts’ average parasite densities and the average parasite densities performed by the Model 2.

To visualize the results, we used a scatter plot (see Figure 5) and to measure the agreement between the two measures, we used the coefficient of determination ($R^{2}$). The results show that there is a high degree of agreement (93%) between the reports made by the Model 2 when compared to the average of the reports made by malaria experts. It is important to mention that the results obtained are statistically significant (*p* < 0.001).

### 5.4. Reading and Reporting Time

**Figure 6:**
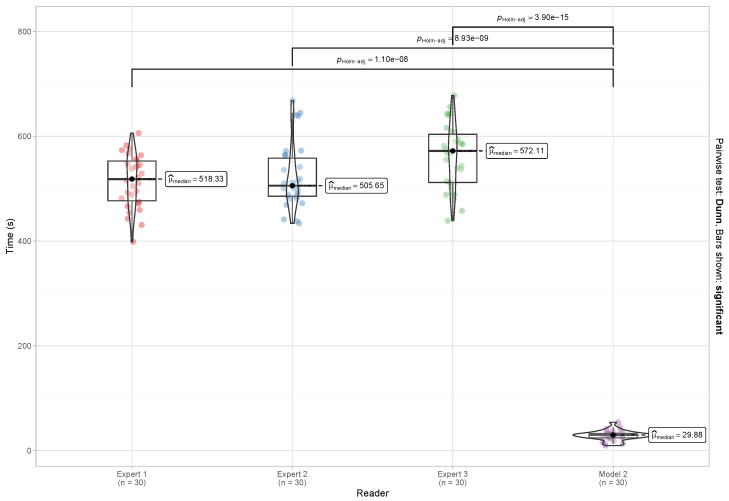
Comparison of the time taken by the experts and the Model 2 to read the 50-image packets.

Time is a key factor in malaria diagnosis; therefore, we wanted to evaluate whether the model trained with augmented data has the ability to report parasitemia counts in a shorter time than humans do. To test this hypothesis, we used the imaging packages from the previous subsection. We measured the time each expert took to read each packet of 50 images. The time taken by Model 2 was also recorded. The Kolmogorov–Smirnov test with the Lilliefors correction showed that the data did not follow a normal distribution. The Kruskal–Wallis test showed that there were significant differences between the time spent by the model and the time spent by the three experts (*p* < 0.001). Figure 6 shows the time spent by each of the experts, the proposed model, and the statistical significance using Dunn’s pairwise comparison test. The results indicate that Model 2 processed packets of 50 images in an average time of 29.88 s. These findings surpass those of the experts, who exhibited slower reading speeds with medians of 518.33 s, 505.65 s, and 572.11 s for Expert 1, Expert 2, and Expert 3, respectively.

## 6. Discussion

In this study, we aimed to develop a clinical decision support system for malaria detection in thick drop images using DL. To achieve this goal, we used different approaches such as data augmentation and the detection of both parasites and leukocytes to report the parasite density as recommended by WHO [2]. Additionally, we measured the image reading time and compared it with a panel of experts in malaria diagnosis.

### 6.1. General Discussion

In the field of AI applied to malaria detection, the availability and quality of the dataset are crucial aspects that directly influence the effectiveness of DL models. The generalization capability and the accuracy of the model are highly dependent on the diversity and quantity of training data [56]. In this context, the present study provides an interesting perspective on data augmentation strategy and its impact on model performance. The fundamental premise that an increase in dataset size leads to improved performance of DL models is supported by the use of the data augmentation process in our study. This involves generating variations of the original images, thereby increasing the diversity of the training set. This approach not only increases the amount of data, but also introduces variability into the training set, allowing the model to learn to recognize a wider range of patterns and features. Figure 4 provides a visual representation of the variations generated through the data augmentation process. This illustration exemplifies how data augmentation can capture subtle differences in the original images, such as changes in illumination, orientation, or size. These variations are crucial in malaria detection, where differences between samples can be subtle but significant.

Our research addresses a fundamental issue in malaria detection: the identification of parasites and leukocytes in thick-drop imaging samples. To achieve this, two detection models were developed and compared using the YOLOv8 algorithm. These models were trained and evaluated on two different datasets: one based on original data and the other on augmented data. First, the model was trained using 222 images in the original dataset, which contained two main classes: parasites and leukocytes. The results obtained were encouraging, with an accuracy of 91% for parasite detection and 90% for leukocyte detection. These findings suggest that the model was able to make robust identifications on this initial dataset. However, the real innovation lies in the augmented data approach. By expanding the dataset to 666 images, the model exhibited even more impressive performance. Accuracy increased significantly, reaching 95% for parasite detection and an outstanding 98% for leukocyte detection. These results highlight the power of data augmentation in improving the performance of the detection model, which may have substantial implications for clinical accuracy and diagnostic capability. Interestingly, along with the improvement in accuracy, an improvement in sensitivity and specificity values was also observed when using the augmented dataset. This suggests that the model was better able to identify both true positives and true negatives, which could translate into improved diagnostic reliability.

The application of AI in the field of malaria detection offers a promising approach to improve accuracy and efficiency in parasitemia assessment. In this study, a comprehensive analysis was conducted using a model trained with augmented data, known as Model 2, which demonstrated superior performance in disease detection as evidenced in Table 1. For parasitemia assessment, a method was employed that considered each packet or set of 50 images as a representation of a patient. This approach simulated realistic clinical assessment, and the packages were provided to both clinical malaria experts and the developed model. The results of this study present an encouraging picture. When comparing the reports generated by the clinical experts with the reports produced by Model 2, a striking concordance is observed. The results reveal a high degree of similarity, reaching 93% agreement. This finding highlights the model’s ability to make detections consistently and accurately compared to human interpretation. Visualization of the results using a scatter plot (see Figure 5) allows us to appreciate the relationship between expert measurements and model predictions. Furthermore, the use of the coefficient of determination ($R^{2}$) as a measure of agreement provides a quantitative basis to support the conclusion that Model 2 is highly consistent with the assessments made by experts in the malaria field. Crucially, the statistical significance of these results, supported by a *p* < 0.001, is noteworthy. This further reinforces the validity and reliability of the conclusions obtained in this study. Taken together, these findings demonstrate the potential of AI, represented by Model 2, to play a crucial role in improving the detection and assessment of parasitemia in malaria patients, which could have a significant impact on the medical care and diagnosis of this disease. However, it is important to continue to investigate and refine these approaches to ensure their effectiveness in real-world clinical scenarios.

The speed of malaria diagnosis has been a critical consideration in the search for better methods to detect and quantify parasitemia. In this study, we addressed this issue by evaluating an AI model trained with augmented data. Our focus was on determining whether this model has the potential to significantly reduce the time required to report parasitemia counts compared to human readout. To test this hypothesis, we employed previously obtained image packets and evaluated the time each expert required to analyze a packet of 50 images. Our results reveal that Model 2 processed the 50-image packets in an average of approximately 30 s, achieving an impressive reading speed of 0.61 s per image. This efficiency far exceeds the readout speeds exhibited by human experts. Specifically, we observed that Expert 1 took on average 10.36 s per image, Expert 2 took about 10.11 s per image, and Expert 3 required approximately 11.44 s per image. These findings suggest a promising advance in the application of AI to malaria diagnosis. The ability of Model 2 to process images and calculate parasite densities in a significantly shorter time than human experts could have a substantial impact on the speed and efficiency of diagnoses. However, it is important to consider that this research also raises questions about practical implementation, the interpretation of results, and possible ethical and technical limitations. Taken together, these results highlight the potential of AI as a valuable tool in the fight against malaria and other infectious diseases.

### 6.2. Training and Validation Loss

**Figure 7:**
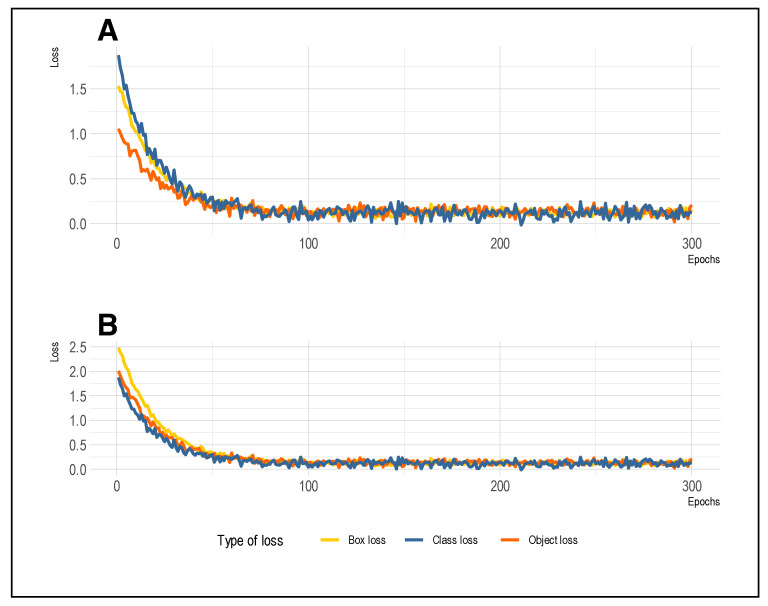
Box, class, and object losses of the training and validation sets. (**A**) represents the losses for the training set and (**B**) the losses for the validation set.

To assess our model’s ability to generalize and prevent overfitting, we examined the box, class, and object losses. Figure 7 illustrates how these losses behave across both the training and validation sets. We observed a notable decline in losses with each increase in epochs, signaling an improved alignment of the model with the data. Furthermore, we found that the box, class, and object losses converge as training advances, indicating the model’s adeptness at capturing pertinent data patterns. This convergence was observed in both the training and validation sets, suggesting that the model was not overfitting the training data and can generalize to new data. This finding is crucial for the accurate detection of parasites and leukocytes in previously unseen images, which reinforces the robustness and clinical utility of the model in real-world applications. Regarding the overestimation of parasite density, instances may occur where the model overestimates the parasite density. For instance, this might happen due to the presence of cell clumps. In scenarios where blood cells or similar elements cluster together, resembling parasites in both shape and color, the model could erroneously identify them as multiple individual parasites. Additionally, staining artifacts resembling parasites may be present in certain images. The model might mistakenly interpret these artifacts as additional parasites, thereby resulting in an overestimation.

### 6.3. Quantitative Comparison with Previous Works

To evaluate the performance of our proposed approach, we conducted a quantitative comparison with studies that used the same dataset. The objective was to perform a fair comparison between models reported in the literature, where the data used in training were the same. Table 2 shows this quantitative comparison in terms of performance metrics for malaria parasite detection. Of the works compared, our model was better in terms of accuracy and sensitivity; however, it had lower values in terms of specificity. Regarding mAP, the values obtained in our study were similar to those reported in the literature. Considering this comparison, we can demonstrate that our model is competent in detecting malaria parasites and that it can be used as a medical decision support system to improve the disease diagnosis process.

### 6.4. Qualitative Comparison with Previous Studies

The diagnosis of malaria through the use of microscopy is a laborious, time-consuming method that requires the knowledge of trained personnel to perform an efficient detection. In the case of new technologies, most systems have the limitation of performing only detection and not the quantification of the parasite, which is necessary to estimate the severity of the disease and administer optimal treatment. Therefore, in this work, we have focused on taking advantage of DL to develop a malaria parasite detection model, which was compared with experts in the field to evaluate its performance. In order to place our study in the context of previous research, we conducted a comparative evaluation using qualitative criteria to understand the advantages and novelty of our approach over other work. Table 3 shows the qualitative criteria used for this comparison.

Several reports in the literature show various studies using AI techniques for malaria diagnostic support [62]. For example, Molina et al. [61] developed a model based on convolutional neural networks. The main contribution of their work is the ability to differentiate parasitized red blood cells from normal red blood cells or from red blood cells with other types of inclusions, such as Howell–Jolly bodies, Pappenheimer bodies, basophilic stippling, and overlying platelets. The study has some disadvantages; initially, the parasite detection is performed on thin blood smears, and the model does not perform the detection of white blood cells, which is necessary to calculate the parasite load. Although the results show the parasitemia in percentage, it is not the one indicated according to WHO protocols, and finally, there is no comparison of the model with readings made by human experts. Yang et al. [23] built a smartphone tool using Iterative Global Minimal Minimal Screening (IGMS) and a convolutional neural network for malaria classification. The model was evaluated on a set of 1819 thick-drop images, obtaining an accuracy of 93.43%. The study reported a parasite detection time of 10 s on a 3024 × 4032 pixel image, and a correlation coefficient of 0.98 between parasites detected by the system and the real data. The approach has the ability to recognize white blood cells; however, the function is used to remove them as a distraction to subsequently generate parasite candidates. The mobile application shows the parasite and WBC count per field but does not perform the overall count for parasitemia. Rosado et al. [14] developed an easy-to-use and adaptable tool for smartphones to detect malaria in thick blood smears using the support vector machine classifier. The model performs the joint detection of white blood cells and *Plasmodium falciparum* trophozoites with an accuracy of 94.9% and 91.8%, respectively. In terms of computational performance, the average detection time recorded was 4.59 s running on a CPU and 44 s on the smartphone. Despite performing white blood cell detection, they are not used for the determination of parasite load, nor do they perform comparison with the method performed by experts. Abdurahman et al. [58] modified versions of YOLOv3 and YOLOv4 to improve small object detection by extending feature scales and adding more detection layers to the neural network. They used thick blood smear images to determine *Plasmodium falciparum* parasites. The best model was achieved by modified YOLOv4 with a mAP of 96.32% and an accuracy of 94.36%. The detection time was 29.60 FPS (frames per second); however, they did not compare the time with experts. The model does not allow the determination of parasite density in the patient because it does not perform quantification and does not detect white blood cells. De Souza Oliveira et al. [24] developed a model based on multilayer perceptron and HVS components. The obtained patches were classified by a deep neural network. Their study focused on the challenge of automated methods for the detection of parasites because they present different shapes and sizes according to the parasite stage. They used four datasets classified according to morphological characteristics, the best results were obtained in the detection of large parasites. The model does not perform white blood cell determination; therefore, it does not calculate parasitemia. The authors also did not focus on evaluating the performance compared to conventional diagnosis. Manescu et al. [26] used convolutional neural networks for the determination and estimation of parasitemia as recommended by WHO. As the training set, they used 169 gross smears and 130 for validation. The results obtained showed an accuracy of 91%. The comparison with the experts was evaluated in different ranges, taking into account low, medium and high parasitemia, finding a correlation index of 95%. In high parasitemias, the approach agreed closely with the human count, but in medium and low parasitemias, the result was overestimated. The study did not evaluate the time taken by the model and the experts to perform the parasite detection, a variable that is of importance because it is a key factor in the diagnosis of malaria.

Our research focused on overcoming the challenges outlined above. We have developed an easy-to-use and adaptive model based on a convolutional neural network, using the YOLOv8 model, in order to optimize accuracy, consistency, and speed in the diagnosis of malaria as a support method for the personnel in charge of the determination of the disease. Our approach uses thick blood smear images for the detection and quantification of parasites and leukocytes. The result obtained is expressed in number of parasites/μL of blood, according to criteria published worldwide by the WHO. In addition, in this study, the validation of the model was carried out by comparing the results of parasite quantifications with the conventional diagnosis, which is the microscopy of thick blood smears performed by experts in the area. A high correlation was obtained between the experts and the developed model. Additionally, our AI-based approach performed parasite detection faster than humans, with a reading speed of one image per second.

## 7. Conclusions

In this research, we have addressed the challenge of malaria detection in thick-drop images by developing a clinical decision support system based on DL. We have explored approaches such as data augmentation and parasite and leukocyte detection to improve diagnostic accuracy and efficiency, following WHO recommendations. The importance of adequate datasets in AI applied to malaria has been highlighted, as model generalization and accuracy depend on the diversity and amount of training data. Our data augmentation approach has proven to be especially valuable in generating variations of original images, which increases the amount and diversity of the training set. This strategy has led to remarkable improvements in the accuracy and performance of the model, demonstrating its ability to recognize a wide range of patterns and features, crucial for malaria detection.

We have developed and compared two detection models using YOLOv8, training them on original and augmented datasets. The results showed that the augmented data approach has led to higher accuracy in parasite and leukocyte detection, with significant implications for diagnostic reliability. The Model 2, trained with augmented data, has been shown to be highly consistent in the detection of malaria compared to assessments performed by clinical experts. This consistency, supported by quantitative and statistical analyses, highlights the potential of AI to improve the efficacy of parasitemia detection in malaria patients. In addition, the Model 2 has demonstrated markedly superior processing speed compared to human experts, suggesting that AI could significantly accelerate malaria diagnosis.

This study shows how AI technologies can be used to diagnose malaria. DL algorithms have remarkable accuracy when analyzing medical images; this could result in earlier and more accurate detection of malaria. This greatly increase diagnostic efficiency by reducing false positives and false negatives. In addition, such models could be available in underserved or isolated locations with inadequate medical infrastructure. In areas with limited access to healthcare, this could extend the reach of medical care and improve the early identification of malaria. The development of an effective model can facilitate its application in other contexts or areas, thus offering enormous potential for global impact in the fight against malaria.

This work has some important limitations that need to be considered. In the field of AI applied to malaria detection, there are important limitations that need to be addressed. One of the key limitations is the availability and quality of the dataset. The effectiveness of DL models is highly dependent on the amount and diversity of training data. Although our study used data augmentation techniques to expand the original dataset, there may still be challenges in the representativeness and diversity of the images. Furthermore, while the results show a significant improvement in accuracy through the use of augmented data, it is important to keep in mind that model performance could be influenced by the quality of the generated images and the possibility of introducing noise or unwanted biases into the training set. Another limitation relates to the assessment of parasitemia and comparison with human experts. Although Model 2 demonstrated remarkable efficiency by processing images in a much shorter time than experts, the interpretation of results and clinical implementation could face additional challenges. The 93% agreement observed between model and expert reports is encouraging, but it is important to consider the potential discrepancies and errors that could arise in real clinical situations.

Another limitation of our study is the lack of model deployment. The deployment of this type of model is difficult for the following reasons: (1) Although DL models have a high degree of accuracy, they often operate as “black boxes”, making it difficult to understand how and why they predict certain things. Patients and medical professionals may have difficulty accepting and trusting it. (2) In many malaria endemic areas, there are limitations related to an adequate technological infrastructure, which makes it difficult to implement AI models for disease diagnosis support. The use of this technology may be hampered by inadequate hardware, Internet access, and skilled manpower. (3) Ethical and privacy issues pose an additional challenge: the use of AI models in the medical setting raises ethical and privacy concerns. It is imperative to ensure the ethical treatment of patient data and the protection of individual privacy, especially in environments where regulations may be absent or loosely enforced.

## Figures and Tables

**Table 1:** Performance of models for malaria parasite and leukocyte detection. Model 1 used the original dataset and Model 2 used the dataset after the augmentation process.

Model	Dataset	Parasite				Leukocyte			
		Accuracy	Sensitivity	Specificity	mAP	Accuracy	Sensitivity	Specificity	mAP
Model 1	Original	0.91	0.91	0.90	0.90	0.90	0.89	0.89	0.91
Model 2	Augmented	0.95	0.94	0.93	0.95	0.98	0.95	0.97	0.97

**Table 2:** Quantitative comparison of our work with previous studies. NR = not reported.

Study	Accuracy	Sensitivity	Specificity	mAP
[23]	0.93	0.93	0.94	NR
[57]	0.89	NR	NR	NR
[58]	NR	0.94	NR	0.96
[59]	NR	0.93	NR	0.94
[60]	NR	0.93	NR	0.66
Our work	0.95	0.94	0.93	0.95

**Table 3:** Qualitative comparison of our work with previous studies.

Qualitative Criteria	Study						
	[61]	[23]	[14]	[58]	[24]	[26]	Our Work
Use of thick-drop imaging	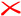	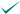	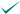	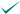	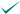	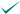	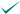
Detection of multiple classes (parasites and leukocytes)	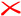	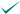	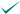	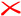	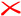	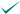	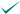
Parasite density calculation according to WHO	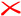	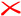	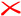	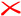	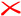	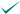	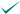
Comparison with clinical experts	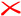	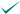	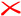	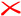	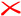	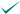	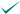
Evaluation of detection time	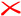	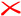	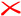	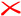	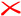	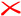	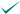
Easy to use and adaptable	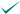	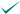	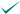	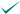	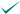	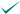	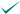

## Data Availability

Data from this research are available upon request.
